# Supplementary material for: Polishing of metal 3D printed parts with complex geometry: Visualizing the influence on geometrical features using centrifugal disk finishing
Source: PLoS One. 2023 Aug 15;18(8):e0289730. doi: 10.1371/journal.pone.0289730 (PMC10427012; doi:10.1371/journal.pone.0289730)
Supplement: Attachment — Submitted filename: Comments_PONE-D-22-29565.docx [file pone.0289730.s001.docx]

Comments

In this paper authors used the centrifugal disk finishing process for finishing of o improve the quality of 3D printed surfaces. The authors designed and 3D printed test parts with different features using selective laser melting, which were coated with a blue metal lacquer prior to polishing. The surface improvement is observed through Mitutoyo SJ-301 Surftest instrument.

1. The authors have not added the image of CDF instrument.
2. Heat treatment parameter need to mention for fabricated structure.
3. Tolerance of Mitutoyo SJ-301 Surftest instrument is not mentioned while measurement of surface roughness.
4. What size of abrasive used in coarse, medium and fine size particles.
5. What was impact of different speed and concentration on finishing quality of surface?
