# Supplementary material for: Polishing of metal 3D printed parts with complex geometry: Visualizing the influence on geometrical features using centrifugal disk finishing
Source: PLoS One. 2023 Aug 15;18(8):e0289730. doi: 10.1371/journal.pone.0289730 (PMC10427012; doi:10.1371/journal.pone.0289730)
Supplement: Attachment — Submitted filename: Response to Reviewers.docx [file pone.0289730.s002.docx]

Dear Editor and reviewers,

We would like to thank you and the reviewers for their efforts in reviewing our work. We carefully went through the reviewers’ comments and created an improved version of the paper, based on their comments. We are resubmitting the revised manuscript, together with this accompanying letter answering in detail to each comment. All the modifications introduced in the revision process are marked up using the “Track Changes” in the text.

Kind regards,

The Authors

**Comments from the editors and reviewers:**

***Reviewer 1***

*The paper focuses on the polishing of SLMed parts by using CDF apparatus. The work shows many lacks in the description of the methodology and the method itself. In following the list of them.*

***Comment 1***

Introduction: some works on the mass finishing applied to SLM parts are neglected. Interesting papers provide basic understanding of the mechanisms and the attainable roughness.

***Authors***

We thank the reviewer for this suggestion, we have added the following paragraph on this topic in lines 61 - 76 in the Introduction:

“The working principle of MF is based on the use of abrasive polishing media, such as ceramic or plastic beads, agitated by mechanical means, often by rotation or vibration. The polishing media, in some cases combined with a liquid medium, comes into contact with the surface of the submerged parts and removes material from the surface by abrasion, as well as impart a polishing effect. Important processing parameters for MF processes are the size of the polishing media, speed of motion, and processing time. Generally, the use of larger sized particles results in a higher material removal rate and a better surface quality (14,15). Prolonged contact with the abrasives in the form of a longer processing time results in the same effect (14,16,17), up to a limit (15). Higher speeds are also associated with higher material removal rates, due to higher forces being applied to the workpieces (16,18,19). The fill level of the container with polishing media was shown to have a negligible effect on material removal rates, higher load levels decreased the chance of contact between the abrasives and workpiece (14,18). The advantages of MF processes are that the process is hands-off, it can process multiple parts at once, and it can be customized to the type of metal and surface finish required.

When the settings of MF processes are properly selected, surfaces with Ra of 0.52 to 5 µm Ra can be obtained (14). …”

***Comment 2***

Material and Methods: since the previous observation, the line 103 should be revised adding information coming from the references.

***Authors***

We thank the reviewer for this suggestion. Due to COVID restrictions at the time, we were unable to use our own facilities, and instead we outsourced the production of the parts to an external company. As such, the specific settings used for the SLM printer, such as hatch distance and laser intensity, were not disclosed since they are proprietary information of the commercial company. As an alternative we gave the surface roughness value, which gives an indication of the initial roughness of the part with which we started. The surface roughness corresponds to values commonly encountered in SLM studies. However, we do not feel comfortable speculating towards the precise settings of the SLM process used. We have clarified in the revised manuscript in lines 113 - 114 the reason for not giving the values:

“The specific print and processing settings used for the test parts were not disclosed by the manufacturer.”

In addition, in lines 177 - 178, we added references to studies with similar surface roughness values:

“The initial surface roughness on the side of the test part was Ra = 9.49 µm before polishing, which is in line with reported roughness values between 2 and 15 µm Ra for metal printed parts (14,28).”

***Comment 3***

Material and Methods: the use of lacquer leaves many doubts about the media to surface interactions at the beginning of the operation. The CDF is a delicate operation with relatively low energy and activation mechanisms (chipping, plastic deformations, microcracking, microfatigue) may be plagued, modified or postponed.

***Authors***

This is indeed true. We acknowledge that the presence of the lacquer influences the results of the CDF process. Therefore, our study was not meant to speculate about the efficiency or suitability of the CDF process, but rather an attempt to gain more insights into the effect of the process on various types of features. The influence of the lacquer on the obtained results depends on many factors, which we feel are outside the scope of this study. Rather, we hope to have shown a practical approach towards gaining useful insights into design guidelines to implement CDF on SLM-ed parts. The above is addressed in lines 147 - 148:

“No additional surface roughness measurements were performed, since the applied lacquer would interfere with the measured roughness values.”

As well as in the section Limitations and Recommendations, lines 259 - 264:

“…the presence of the lacquer on the part will have influenced the effectiveness of the polishing process to some extent. Although the lacquer is less hard than the stainless steel of which the parts are made, this is still an additional layer that needs to be polished away. Therefore, it is an effective measure of visualizing the polishing patterns, but less suitable to determine the actual efficiency of the polishing process with regards to the surface roughness and the material removal rate.“

In addition, we clarified line 94 in the Introduction, and added lines 125-127 in Materials and Methods:

“It should be noted that since the lacquer influences the effectiveness of CDF, this process will result in less improvements in surface finish than otherwise can be obtained.”

***Comment 4***

Material and Methods: authors didn’t provide an experimental plan to find polishing parameters such as: media shape size and material, compound amount and type, rotational speed, water flow, filling percentage, etc. The manufacturer information and experience are generally related to 316L parts fabricated via traditional technologies and different results are expected on the SLMed material completely different in microstructure.

***Authors***

We agree with the reviewer, however we do feel that an experimental plan to find the optimal polishing parameters is out of the scope of this study. The goal of our study was not to find the optimal polishing parameters for the test part, or to obtain a perfectly polished part, but rather to collect information to indicate the effect of CDF on certain features of complex parts. The recommended manufacturer settings have been applied in preliminary tests, however were not optimized for this specific test part.

We clarified this in the manuscript by adding the following in lines 95 - 97 and 101 -102 in the Introduction:

“Therefore, in this study, we investigate the influence of centrifugal disk finishing (CDF), a common MF process, on the geometrical features of a complex, 3D printed part, in order to obtain information that can be used in the design process. *Since the effectiveness of CDF on surface improvements has already been proven, we did not optimize the settings of CDF for our part. Instead, we investigate how geometrical features are altered as a result of the process, and which types of features cannot be accessed by CDF.* In order to do so, we designed an experiment in which a 3D printed test part was coated with a blue marking lacquer for metals, before undergoing a number of different polishing steps. After polishing, the remaining lacquer gives a visual pattern of the surfaces on the part to which the polishing media had access. *The results led to a number of design guidelines that can be of help during the design process of SLM parts*.”

In Limitations and Recommendations, lines 264 - 268 we added the following:

“Additional research to optimize the processing settings of CDF could improve the obtainable results, for instance by comparing the influence of different rotational speeds and media sizes on the geometrical features. We recommend that in order to optimize the processing settings for a specific design, test parts with representative features of the final design should be used (29).”

***Comment 5***

Results and discussion: The previous observation can explain the Ra results of this work. The obtained values after the polishing reveal that the selected processing parameters are not really efficient with respect to other mass finishing operations applied to the same SLM material.

***Authors***

This is indeed true, however we cannot discount the effect of the influence of the lacquer, which had to be polished away first, and led to a worse surface roughness than otherwise could have been obtained. Many other studies, as noted in Comment 1, have shown the potential of CDF with optimum parameters. We are fully aware that the approach shown in this study would not lead to optimal processing settings due to the influence of the lacquer, but it was used for the generation of design guidelines that can be applied to parts that should be polished using CDF. We refer to our response to Comment 3 and 4 as to the alterations we made in the manuscript to clarify this point.

***Comment 6***

Results and discussion: The assessment at line 190 should be verified.

***Authors***

We thank the reviewer for this observation, we have nuanced the statement (line 209) to:

“This is in line with other research suggesting larger particles are responsible for most of the material removal (14,15).”

***Comment 7***

Material and Methods: The experimental campaign is poor and extraction at intermediate times is general requested for a deeper investigation of the surface evolution. The image processing technique lacks in repeatability with the following issues. The using of a Photoshop algorithm does not allow a clear understanding of the RGB channels selection criteria. Moreover, when a color selection is adopted, particular care must be paid on the light source which may modify the radiation reflection spectrum and have local and surrounding undesired effects on the under-detection feature. Finally, no indication or motivation is provided about the selection of the threshold in Matlab.

***Authors***

We agree with the reviewer that extraction at intermediate times is generally requested for a deeper investigation of the surface evolution, however this was not the goal of our study. Rather we wanted to illustrate the effects of CDF on geometrical features, since many other studies have attributed themselves to investigating the surface evolution, as pointed out in Comment 1.

The image processing technique is indeed not perfect. However, we took care to ensure the process is repeatable by giving as many specifications as possible. The Photoshop process used to extract the blue tones is based on an algorithm built into the software, we have not used the option that lets us select the RGB channels manually. Therefore, for photos taken in similar lighting circumstances results of the tool will be comparable and the algorithm will be repeatable. We have clarified this in Line 160 - 161:

“The images were processed in Photoshop (Adobe Inc., USA) to extract only the blue tones, using the ‘Color Range’ tool and then selecting ‘Blues’, which automatically selects the blue pixels in the image.”

For the photos, we took care that the lighting and source were the same for each image, although we can indeed not guarantee that this result is 100% reproducible. Since the lighting conditions were the same for all images, we feel comfortable comparing the results with each other, as potential reflection effects will occur in the same amount for all images. We have added the lighting conditions in which the images were taken in Line 158.

The threshold in Matlab was used because of previous results obtained with similar scripts, for which we have now added the references in Line 163.

***Reviewer 2***

*In this paper authors used the centrifugal disk finishing process for finishing of o improve the quality of 3D printed surfaces. The authors designed and 3D printed test parts with different features using selective laser melting, which were coated with a blue metal lacquer prior to polishing. The surface improvement is observed through Mitutoyo SJ-301 Surftest instrument.*

***Comment 1***

The authors have not added the image of CDF instrument.

***Authors***

We thank the reviewer for this suggestion, we have added the image in Figure 2c.

***Comment 2***

Heat treatment parameter need to mention for fabricated structure.

***Authors***

Unfortunately, since we had to rely on a commercial company due to COVID restrictions at the time, we do not have the exact heat treatment parameters, since this is proprietary information of the company. We clarified this in Line 113 - 114:

“The specific print and processing settings used for the test parts were not disclosed by the manufacturer.”

***Comment 3***

Tolerance of Mitutoyo SJ-301 Surftest instrument is not mentioned while measurement of surface roughness.

***Authors***

We thank the reviewer for this suggestion, we have added the tolerance in line 116:

“The surface roughness of the flat side on one of the test parts was measured (Mitutoyo SJ-301 Surftest, Mitutoyo Corporation, Japan) with a tolerance of 0.01 µm …”

***Comment 4***

What size of abrasive used in coarse, medium and fine size particles.

***Authors***

The polishing particles are shown in Figure 2d with a ruler, for clarity we have also added the sizes in Table 1, row 3:

|  | **Coarse step** | **Medium step** | **Fine step** |
| --- | --- | --- | --- |
| Polishing media | DZS 6/6 | KM 10 and PM 10 | ZSP 3/5 and DZP 3/3 SK |
| *Size* | *6 x 6 mm* | *10 x 10 mm and 10 x 10 mm* | *3 x 5 mm and 3 x 3 mm* |
| Compound | SC15 | SC15 | SC5 |
| Speed | 280 rpm | 260 rpm | 220 rpm |
| Water flow | 10 L/h | 10 L/h | 10 L/h |
| Water concentration | 3% | 3% | 3% |
| Type | Ceramic-bonded | Plastic-bonded | Ceramic-bonded |

***Comment 5***

What was impact of different speed and concentration on finishing quality of surface?

***Authors***

In this study, we did not research the optimum processing parameters or their influence on the surface quality. Rather, we focused on the influence of the different steps on the geometrical features. However, previous research has indicated the influence of various process parameters on the surface quality. We have added this information in Lines 61 – 76:

“The working principle of MF is based on the use of abrasive polishing media, such as ceramic or plastic beads, agitated by mechanical means, often by rotation or vibration. The polishing media, in some cases combined with a liquid medium, comes into contact with the surface of the submerged parts and removes material from the surface by abrasion, as well as impart a polishing effect. Important processing parameters for MF processes are the size of the polishing media, speed of motion, and processing time. Generally, the use of larger sized particles results in a higher material removal rate and a better surface quality (14,15). Prolonged contact with the abrasives in the form of a longer processing time results in the same effect (14,16,17), up to a limit (15). Higher speeds are also associated with higher material removal rates, due to higher forces being applied to the workpieces (16,18,19). The fill level of the container with polishing media was shown to have a negligible effect on material removal rates, higher load levels decreased the chance of contact between the abrasives and workpiece (14,18). The advantages of MF processes are that the process is hands-off, it can process multiple parts at once, and it can be customized to the type of metal and surface finish required.”
