# Supplementary material for: Polishing of metal 3D printed parts with complex geometry: Visualizing the influence on geometrical features using centrifugal disk finishing
Source: PLoS One. 2023 Aug 15;18(8):e0289730. doi: 10.1371/journal.pone.0289730 (PMC10427012; doi:10.1371/journal.pone.0289730)
Supplement: Attachment — Submitted filename: Response to Reviewers 2.docx [file pone.0289730.s003.docx]

Dear Editor and reviewers,

Hereby we submit our revised manuscript in response to the reviewers’ comments. We are resubmitting the revised manuscript, together with this accompanying letter answering in detail to each comment. All the modifications introduced in the revision process are marked up using the “Track Changes” in the text. Hopefully the responses below clarify any confusion about this study.

Kind regards,

The Authors

**Comments from the editors and reviewers:**

***Reviewer 1***

***Comment 4 response:***

*I don't agree with the answer t ocomment #4: since a manufacturing process is applied, it is important to deepn how the processing paramaters the author selected, both in term of factors and levels, affect the results; the sentence "we investigate the influence of centrifugal disk finishing (CDF), a common MF process, on the geometrical features of a complex, 3D printed part," highligths the use of a traditional technique on a non traditional part (AMed material); I am not requesting an optimization, rather the investigation the autthors mention. This point could help to provide observation on comment #5.*

***Authors***

The settings that were recommended by the manufacturer were in this case for 3D printed parts, not for traditionally manufactured parts. This recommendation was based on the coarse nature of unfinished SLM’ed parts, and was tested on a part before starting this test. There are still many more investigations necessary to determine the effect of all processing parameters on AM-ed parts. Also, the selection of processing parameters depends on the required result, within the requirements of the design. In this case that can explain the less effective result compared to other mass finishing operations, as was pointed out in comment 5.

Since there is already a lot of research into how the process parameters of CDF influence the surface characteristics for SLM’ed parts, we chose fixed process parameters and focused on a few differences, i.e. duration of polishing time and coarseness of the media, to investigate which effect these have on the geometrical features. We clarified the above and explain that we focus on the coarseness of the media and the total polishing duration (lines 128-132):

“In a preliminary test, a polishing schedule consisting of three steps of 120 minutes with coarse, medium, and fine polishing media was tested on a simple part, as advised by the manufacturer of the CDF *for parts produced using SLM*. In Table 1, the details of the used process steps are given, for each step fresh media was used. *We investigated the influence of the coarseness of the media and the total duration of polishing on the geometrical features*, by dividing the twelve parts into four groups of three that were polished in different polishing steps, as shown in Table 2.”

We also addressed this in the recommendations and limitations section (lines 261-264):

“Additional research into other process parameters of CDF, such as rotational speed, is required to provide insight into their effect on geometrical features. We recommend that in order to optimize the processing settings for a specific design, a test part can be used with representative features as used in the final design (29).”

***Response Comment 7****: the authors should check what is provided by software they cannot control. In a scientific research the results must be sure as well as the methods to retrieve them.*

***Authors****:*

We believe there is no part of the software that is out of our control, in fact, the way in which we used the software ensures that the results are the same for all images and can be compared. Adobe Photoshop is a common tool used in image processing, similar approaches are for instance used in references [1-3] below (but many more can be found), which show the Color Range command is reliable in selecting a specific range of colour for the purpose of image processing. We added a brief explanation on the working of the color range command in the Methods section (lines 156-159), although we feel expanding on this method more is out of scope for this study:

“The images were processed in Photoshop (Adobe Inc., USA) to extract only the blue tones. An RGB image contains three channels: red, green, and blue. The ‘Color Range’ command allows the user to select pixels of an image based on their colour channel. By selecting ‘Blues’ within the Color Range command, only pixels within the blue channel were selected.”

*[1] Dahab et al. (2004). Digital quantification of fibrosis in liver biopsy sections: Description of a new method by Photoshop software. Journal of Gastroenterology and Hepatology, 19(1), 78–85*

*[2] Chen, Li, and Gao (2018). The Colony Count Based on Image Processing Using Matlab and Photoshop. Advances in Computer Science Research, volume 80*

*[3] Stewart, A. M., Edmisten, K. L., Wells, R., & Collins, G. D. (2007). Measuring Canopy Coverage with Digital Imaging. Communications in Soil Science and Plant Analysis, 38(7-8), 895–902.*
