# Supplementary material for: Polishing of metal 3D printed parts with complex geometry: Visualizing the influence on geometrical features using centrifugal disk finishing
Source: PLoS One. 2023 Aug 15;18(8):e0289730. doi: 10.1371/journal.pone.0289730 (PMC10427012; doi:10.1371/journal.pone.0289730)
Supplement: Attachment — Submitted filename: Response to Reviewers 3.docx [file pone.0289730.s004.docx]

Dear Editor and reviewers,

Hereby we submit our revised manuscript in response to the reviewers’ comments. We are resubmitting the revised manuscript, together with this accompanying letter answering in detail to each comment. All the modifications introduced in the revision process are marked up using the “Track Changes” in the text.

Kind regards,

The Authors

**Comments from the editors and reviewers:**

***Reviewer 1***

*Comment #1: I invite again the authors to motivate the selection of the processing parameters (they told the manufacturer suggested them). In particular some indications must be given for the selection of the geometry which is different for the three levels of charge and obviusly they will affect the contribution to the outcomes.*

***Authors***

The selection of the processing parameters was indeed based on the suggestion of the manufacturer, as we explain in the manuscript. Therefore, we do not have any more information to provide on the selection of the geometry of the charge. For reference, the following studies have used CDF on relatively complex AM parts. Soja et al. [1] used a ceramic angle cut tristar in size 10/12 mm with a runtime of 4 hours to reduce the Ra of their SLM parts from 3.5 – 19.4 to 2.9 µm. Kaynak et al. [2] reduced the Ra from 7 µm to Ra of 2.7 µm after 4 hours with ceramic abrasive particles of undisclosed shape and size. Nicolas et al. [3] used a polymer pyramid of 15 mm and ceramic angle cut triangle in 10/15 mm with a runtime of 1 hour for aluminum and 30 hours for titanium to obtain an Ra of 4.43 µm, down from 10.6, and 3.33 µm, down from 16.3, respectively. Fan et al. [4] used triangular ceramic media of 4/10 mm to obtain an Ra of approximately 4.5 µm after 30 minutes. Lesyk et al. [5] used spherical charge with a diameter of 3 mm for 4 hours and decreased surface roughness by 20%. None give a substantial consideration into the choice of polishing media, and we speculate that the choice is made on a trial-and-error basis or from experience, as is the case for us.

It can be seen from these studies that there is a great variety in the type and shape of abrasive media chosen, and our choice of media is a combination of the types mentioned above. Consideration of the choice of media depends on the size, shape, and weight of the media, however this cannot be seen separately from other process parameters such as packing of the barrel, speed, type of compound, processing time etc. An investigation into the influence of the polishing media on small, complex parts such as ours would warrant an entire study on its own, and would divert the scope of our current study too much.

We provided the following argumentation in the manuscript in lines 128 – 135:

“Generally speaking, larger abrasive media will be more effective on the external surfaces of the part and have a faster cut rate, while smaller media are able to reach into the interior regions and small features (11,26). The size of the abrasive media is also important to keep the submerged parts separate from each other and prevent them from clashing (13). The shape of the abrasive media should be chosen in such a way that it permits access to all surfaces of the part (13). Based on preliminary tests and advise of the manufacturer of the CDF, we settled on a polishing schedule consisting of three steps of 120 minutes with coarse, medium, and fine polishing media with different shapes.”

In the discussion, we added the following elaboration in line 269-270:

“Additional research into other process parameters of CDF, such as rotational speed, and abrasive media shape and size, is required to provide insight into their effect on surface finish as well as geometrical features.”

*Comment #2: Please detail the color selection (numerically which rules is given to select the blue color) and the sensitivity of the procedure to the lighting by providing a sort of calibration which considers the amount of pixels after im2bw function.*

***Authors***

We ran an image with an RGB blue pixel scale ranging in color from 0 to 255, divided in 10 increments, through the Matlab script. The Matlab script was used to calculate the amount of pixels after the im2bw function, which amounted to 80%, corresponding to the 0.8 threshold. We ran this image through the same Photoshop process using the Color Range command and then again through the Matlab script, which resulted in 50% black, indicating that this command includes 62.5% of blue pixels considering the 0.8 threshold. For calibration of the lighting, we photographed one fully coated sample and one fully uncoated sample with the microscope. The uncoated sample amounted to a 0% black output. The coated sample amounted to a 94.7% black output. The difference can be attributed to highlights due to reflection of light which show up as white on the image. However, it should be noted that both coated and uncoated highlights show up white, and therefore the difference cannot be detected, and, since highlights are caused by peaks on the surface, they are more likely to be polished and thus uncoated. Therefore, we did not correct for this value.

We added the following in the limitations and recommendations, line 266-268:

“The image analysis method employed in this study is useful for comparison within batches, but due to sensitivity to lighting the absolute results may vary in different conditions.”
